# Novel Insights Into Peptide‐Calcium Chelates From *Lentinula edodes*: Preparation and Its Structure, Stability, and Calcium Transport Analysis

**DOI:** 10.1002/fsn3.4731

**Published:** 2025-01-07

**Authors:** Haofeng Gu, Lei Liang, Yang Wei, Jia Hao Wang, Wanning Ma, Yuyu Fu, Dan Fan, Wanxiang Gao, Jiayao Yang, Xinyu Zheng, Tingshu Chen, Yuexin Chen

**Affiliations:** ^1^ School of Modern Agriculture & Biotechnology AnKang University Ankang China; ^2^ Guohua Agriculture and Forestry Technology Development Co. LTD Xunyang China; ^3^ Department of Food Science and Engineering, School of Agriculture and Biology Shanghai Jiao Tong University Shanghai China

**Keywords:** calcium‐binding mechanisms, *Lentinula edodes*, microstructure, optimization, peptide‐Ca chelate

## Abstract

Peptide‐Ca chelates are innovative calcium supplements. *Lentinula edodes* possesses nutritional advantages for preparing calcium‐binding peptides (CBPs), although there are limited studies on this subject. Therefore, this paper investigated the optimal condition for preparing *Lentinula edodes* CBPs and *Lentinula edodes* peptide‐calcium chelates (LP‐Ca), along with analyzing their microstructure, calcium‐binding mechanisms, stability, and calcium transporting efficacy. The optimal protease and hydrolysis time for preparing CBPs were neutral protease and 3 h, respectively. The optimized parameters for LP‐Ca preparation were as follows: pH9, time 50 min, mass ratio of peptide/CaCl_2_ 5:1, and temperature 65°C. The chelates contain 4.23% ± 0.01% Ca. After chelation, Glu, Asp, Lys, Ser, His, and Cys were enriched. LP‐Ca possessed a rough and porous structure, exhibiting a pronounced calcium signal. —COO—, C=O, and N—H groups were contributed to the chelation, with calcium primarily existing in an amorphous form. LP‐Ca exhibited enhanced thermal stability and retained most of the calcium (62.33% ± 4.51%) after digestion, and calcium transportation was enhanced in the LP‐Ca group (9.57 ± 0.60 μg). Collectively, LP‐Ca are studied for the first time and the study is of great significance for developing novel calcium supplements.

## Graphical abstract

*Lentinula edodes* peptide‐Ca chelates (LP‐Ca) were prepared. The chelates possessed a rough, loose, and porous structure. —COO—, C=O, and N—H groups were involved in the chelation of LP‐Ca. LP‐Ca exhibited good thermal stability and retained most of Ca after digestion. LP‐Ca exhibited promoting effects on calcium transport by Caco2 cells.

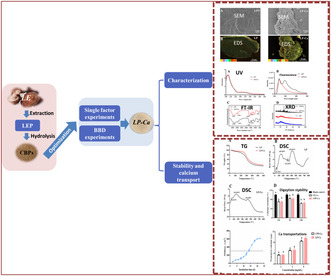

**Abbreviations**
BBD: Box–Behnken design
CBPs: calcium‐binding peptides
CBR: calcium‐binding rate
DSC: differential scanning calorimetry
EDS: energy‐dispersive spectroscopy
FBS: fetal bovine serum
FT‐IR: fourier transform infrared spectrometer
GD: gastric digestion
GID: gastrointestinal digestion
HD: hydrolysis degree
ID: intestinal digestion
LP‐Ca: *Lentinula edodes* peptide‐calcium chelates
LPN: *Lentinula edodes* peptides released by neutral protease
NEAA: nonessential amino acid
SEM: scanning electron microscopy
TEER: transepithelial electrical resistance
TG: thermogravimetric
UV: ultraviolet–visible
XRD: X‐ray diffraction

## 1 Introduction

Calcium is an indispensable nutrient for people and a critical component of bones, teeth, and other tissues (Shlisky et al. 2022). The recommended intake of calcium for adult is 800 mg/day. It also participates in many physiological activities including nerve signaling and hormone regulation (Andreas et al. 2011). Calcium deficiency can lead to cramps, osteoporosis, developmental delays, rickets, etc. (Shlisky et al. 2022). Therefore, adequate calcium intake is essential for maintaining human health. However, Ca deficiency is currently widespread, particularly among pregnant women, children, and the old (Shlisky et al. 2022). In China, more than 90% of people do not get enough Ca. The mainstream calcium supplements on the market include ionic calcium supplements (e.g., CaCO_3_) and inorganic calcium (e.g., calcium gluconate) (Gu et al. 2024). However, these calcium supplements have several shortcomings, such as forming insoluble substances with phytic acid in the intestinal tract, which reduces calcium bioavailability and causes constipation (Wongdee et al. 2019). They can also cause adverse stimuli to the gastrointestinal tract, leading to side effects (Lin et al. 2024). Therefore, it is necessary to develop Ca supplements with high calcium bioavailability and minimal side effects. Peptide‐Ca chelates are complexes formed through the chelation of peptides and Ca^2+^. The complexes offer several advantages: the chelation of calcium by peptides protects Ca^2+^ from interference by phytic acid, thereby improving calcium bioavailability (Lin et al. 2024); Additionally, intestinal epithelial cells can specifically recognize small peptides, allowing the chelated calcium to be directly transported into the cells using the peptides as “carriers,” thus enhancing calcium absorption efficiency (Malison, Arpanutud, and Keeratipibul 2021). Therefore, peptide‐Ca chelates are considered as promising calcium supplements.

In developing peptide‐Ca chelates, it is crucial to prepare CBPs with a high calcium‐binding rate (CBR). From a safety perspective, food‐derived proteins are the best choice for CBR preparation. A variety of peptide‐Ca chelates have been developed using different food proteins, including milk proteins, fish proteins, soy proteins, etc. (Wu, Hu, et al. 2024). *Lentinula edodes* is a medicinal and edible mushroom that contains more than 20% protein and is a promising material for preparing functional peptides such as antihypertensive (Paisansak et al. 2021) and hypoglycemic peptides (Zhang et al. 2023). Notably, Glu and Asp are the two most abundant amino acids in *Lentinula edodes*, at 97.82 ± 9.92 mg/g and 35.65 ± 1.30 mg/g, respectively (Davila and Du 2023). These amino acids display strong affinities for Ca^2+^, providing binding sites in many peptide‐Ca chelates (Wu, Hu, et al. 2024). Thus, *Lentinula edodes* has a nutritional advantage for preparing peptide‐Ca chelates. However, LP‐Ca have sparsely been studied.

Therefore, this paper investigates the preparation conditions of *Lentinula edodes*–derived CBPs and optimizes the preparation of LP‐Ca. The microstructure and calcium distribution of LP‐Ca were analyzed using scanning electron microscopy (SEM) and energy‐dispersive spectroscopy (EDS). The calcium‐binding mechanisms of LP‐Ca were elucidated by ultraviolet–visible (UV), infrared spectroscopy (FT‐IR), and X‐ray diffraction (XRD). Additionally, thermal and gastrointestinal digestive stability of LP‐Ca were evaluated. Finally, its effect on promoting calcium transport was analyzed using a monolayer cell model. This is the first report of peptide‐Ca chelates derived from *Lentinula edodes*, which is of great significance for developing novel calcium supplements.

## 2 Materials and Methods

### 2.1 Materials

*Lentinula edodes* was brought from supermarket in Ankang (Shaanxi, China). Neutral protease (100 U/mg), CaCl_2_, papain (800 U/mg), trypsin (2500 U/mg), fetal bovine serum (FBS), and pepsin (3000 U/mg), were brought from Longxinda Reagent Company (Xi'an, China). Caco2 cells, DEME medium, CCK8 kit, protamex (120 U/mg), trans‐well plate, and nonessential amino acid (NEAA) were brought from Fuxiang Biotechnolgy Co. LTD. (Nanjin, China). Alkaline protease (200 U/mg) and penicillin–streptomycin mixture were brought from Jinrun Biotechnology Company (Wuhan, China).

### 2.2 Extraction of *Lentinula edodes* Protein

*Lentinula edodes* powder (40 g) was dissolved in distilled water (1600 mL) with ultrasound assistance (700 w, 10 min) by an ultrasonic dispersion instrument (JY92‐IIDN, SCIENTZ). After adjusting the solution pH to 10, *Lentinula edodes* protein was extracted (2 h, 65°C). The extracting solution was centrifuged (4000 r/min, 10 min) to collect the supernatant. The *Lentinula edodes* protein was precipitated at a pH of 4.3 for 3 h, followed by centrifugation (6000 r/min, 10 min). The precipitated protein was obtained and freeze‐dried.

### 2.3 Optimization of Preparing CBPs From *Lentinula edodes*

*Lentinula edodes* protein solution (40 mg/mL) was prepared using deionized water. Different enzymes were added (1 × 10^5^ U/protein) to produce CBPs for 3 h. The conditions for the used proteases are listed below: trypsin (37°C, pH6.5), papain (50°C, pH7.5), alkaline protease (55°C, pH8), protamex (55°C, pH7), pepsin (37°C, pH2), and neutral protease (50°C, pH7). *Lentinula edodes* peptides released by neutral protease (LPN), which had the highest CBR, were selected. Furthermore, the hydrolyzing time (0, 0.5, 1, 2, 3, and 4 h) of neutral protease was optimized using CBR as an indicator.

### 2.4 Optimization of the Preparation of LP‐Ca

The preparation conditions for LP‐Ca were optimized using single‐factor and Box–Behnken design (BBD) experiments (Gu et al. 2023). Using CBR as an indicator, single‐factor experiments were conducted by varying one factor while keeping the other three constant. The basic reaction conditions were as follows: temperature 55°C, time 50 min, pH8, and mass ratio of peptides/CaCl_2_ 2:1. Briefly, the LPN solution (10 mg/mL) was reacted with CaCl_2_ at temperatures ranging from 35°C to 85°C and pH (4–9) for 20–70 min, with the mass ratio of peptides/CaCl_2_ ranging from 0.5:1 to 5:1. Subsequently, BBD experiments were conducted using CBR as a dependent variable and four factors (A‐pH, B‐time, C‐mass ratio of peptides/CaCl_2_, and D‐temperature) as independent variables. The levels of these variables are shown in Table 1.

**TABLE 1:** Results of response surface experiments.

Number	A, pH	B, Time (h)	C, Mass ratio of peptide/CaCl_2_	D, Temperature (°C)	CBR (%)
1	8	40	4	55	60.98 ± 0.13
2	8	50	3	55	56.61 ± 0.25
3	8	30	4	45	58.76 ± 0.42
4	8	30	4	45	55.78 ± 0.51
5	9	40	3	55	48.82 ± 1.05
6	7	50	4	55	57.56 ± 0.22
7	7	40	5	55	55.58 ± 0.63
8	9	30	4	55	60.90 ± 0.57
9	9	50	4	55	64.05 ± 1.08
10	7	30	4	55	59.61 ± 2.16
11	9	40	4	65	77.70 ± 0.79
12	7	40	3	55	41.02 ± 0.62
13	8	40	3	65	46.20 ± 0.59
14	8	30	3	55	36.66 ± 0.88
15	8	40	5	45	63.35 ± 0.69
16	8	40	4	55	61.44 ± 1.24
17	8	30	4	65	58.97 ± 0.88
18	9	40	4	45	64.73 ± 1.13
19	8	50	4	65	67.94 ± 0.34
20	9	40	5	55	81.64 ± 2.32
21	8	40	5	65	74.92 ± 0.44
22	8	40	4	55	59.06 ± 1.59
23	8	40	4	55	65.18 ± 0.52
24	7	40	4	45	61.26 ± 0.86
25	8	40	4	55	62.70 ± 1.17
26	8	50	5	55	78.33 ± 0.30
27	8	50	4	45	61.90 ± 2.54
28	8	40	3	45	54.22 ± 0.91
29	7	40	4	65	67.55 ± 0.36

### 2.5 Measurement of CBR

CBR was determined using our previously established method (Gu et al. 2024). Briefly, LP was reacted with CaCl_2_ at 50°C for 40 min. The mass ratio of peptides/CaCl_2_ and pH were 4:1 and 8, respectively. LP‐Ca were precipitated by a 10‐fold volume of absolute ethanol. The bound Ca in LP‐Ca and the total amount of Ca added in the reaction were determined. CBR was calculated using the formula (1):

(1) $$\mathrm{CBR}\;\left(\%\right)=\frac{\text{Content of the bound}\;\mathrm{Ca}}{\text{Total content of}\;\mathrm{Ca}}\times 100$$

### 2.6 Determination of Hydrolysis Degree (HD) and Amino Acid Content

HD was measured using the previous method (Gu et al. 2024). Briefly, a solution of *Lentinula edodes* protein hydrolysates (0.25 mL) was diluted by adding a sodium dodecyl sulfate agent (12.25 mL, 10 mg/mL). The phosphate solution (200 mmol/L, 1 mL, pH8.2) was pippeted into the mixture, followed by adding trinitrobenzene sulfonic acid (0.1 g/100 g, 1 mL). The reaction occurred in an opaque tube (50°C, 1 h). HCl (2 mL, 100 mmol/L) was pippeted into the tube, and the absorbance was tested at 420 nm. The standard curve (*y* = 1.301x + 0.024, *R* ^2^ = 0.99) was established using L‐Leu.

The amino acid content of LPN and LP‐Ca was measured using the traditional method (Gu et al. 2024). Briefly, LPN and LP‐Ca were treated with HCl (10 mL, 6 mol/L) under nitrogen protection for 24 h. The amino acid contents of the two samples were determined using an amino acid analyzer (Biochrom 30, Biochrom, England).

### 2.7 Characterization of LP‐Ca

#### 2.7.1 Microstructure and EDS Analysis

The microstructure and element profile of LPN and LP‐Ca were assayed using an SEM equipped with EDS (ZEISS, Germany) (Wang et al. 2020). Briefly, the two samples were sprayed with gold, and the microstructure image and element distribution were determined under 3 kV.

#### 2.7.2 UV and Fluorescence Analysis

Briefly, LPN and LP‐Ca solutions (1 mg/mL) were prepared using ultrasound (500 W) (Wu, Wang, et al. 2024). The two samples were scanned by a UV spectrophotometer (UV759, YouKe, China) from 190 to 400 nm. For the fluorescence scanning assay, the samples were placed in a quartz cuvette and analyzed using a fluorescence spectrophotometer (RF‐6000, Shimadzu, Japan). The excitation and emission wavelength were 295 nm and 200–500 nm, respectively.

#### 2.7.3 FT‐IR and XRD Assay

Briefly, the sample (4 mg) was ground with KBr (400 mg), and the prepared pellet was scanned from 4000 to 400 cm^−1^ using an FI‐IR (FTIR‐650S, Tianjin Gangdong, China) (Wu, Wang, et al. 2024). The XRD spectra of the sample were obtained using an X‐ray diffract meter (Bruker, Germany).

#### 2.7.4 TG and DSC Assay

Briefly, the sample was placed in an aluminum crucible, and TG and DSC assay were conducted using a simultaneous thermal analyzer (TGA/DSC2, METTLER TOLEDO, Switzerland) under an atmosphere of N_2_. The temperature range was 10°C–800°C (Xiang et al. 2022).

### 2.8 Simulated Digestion Stability Assay

A simulated digestion stability assay was conducted following a previous method (Wu et al. 2022). Briefly, during the gastric digestion (GD) stage, pepsin was added to the LP‐Ca solution (0.5 mg/mL) with a weight ratio of 1:50 (enzyme/chelates). The digestion was conducted for 2 h (37°C, pH2). In the intestinal digestion (ID) stage, pepsin was replaced by trypsin, and the digestion was conducted at pH7. For gastrointestinal digestion (GID), the sample was sequentially treated with GD and then ID. The reserved HP‐Ca were precipitated following the method in Section 2.5. The calcium retention rate of the sample at each stage was calculated using formula (2). CPP‐Ca and deionized water were taken as positive control and blank control, respectively,

(2) $$\text{Calcium retention rate}\;\left(\%\right)=\frac{\text{Amount of calcium in retained}\;\mathrm{LP}-\mathrm{Ca}\;}{\text{Amount of calcium in}\;\mathrm{LP}-\mathrm{Ca}}\times 100$$

### 2.9 Study on Calcium Transport Promoting Effects

#### 2.9.1 Cell Viability Assay

Caco2 cells were cultured in the DMEM medium at 37°C. The medium was supplemented with FBS (15%), penicillin–streptomycin (1%), and NEAA (1%). Cells were seed into a 96‐well plate (6000 cells/well) and grew for 24 h. Different amounts of LP‐Ca (0–32 mg/mL) were then added to each well, and the treatment was sustained for 24 h. Cell viability was analyzed by the CCK8 kit.

#### 2.9.2 Calcium Transport Assay

The effect of LEVEIHA‐Ca on calcium transport was evaluated according to the previous method (Wu et al. 2022). Briefly, Caco‐2 cells were seeded on inserts of a 12‐well trans‐well plate (Corning Costar, USA) and cultured for 3 weeks. When TEER of the cell monolayer was up to 300 Ω, the model was used to evaluate calcium transport. LEVEIHA‐Ca (0.5 mL, 2–8 mg/mL) was pipetted onto the apical side, and HBSS (1.5 mL) was pipetted to the basal side. The amount of transported calcium was measured after 2 h. CPP‐Ca was used as a positive control.

### 2.10 Statistical Analysis

All experiments were performed three times, and the results were analyzed by SPSS 20 (IBM Corporation, USA) with a significance level *p* < 0.05. BBD results were analyzed by Design‐Expert.

## 3 Results and Discussion

### 3.1 The Influence of Proteases and Hydrolytic Time on the CBR of LP

**FIGURE 1:**
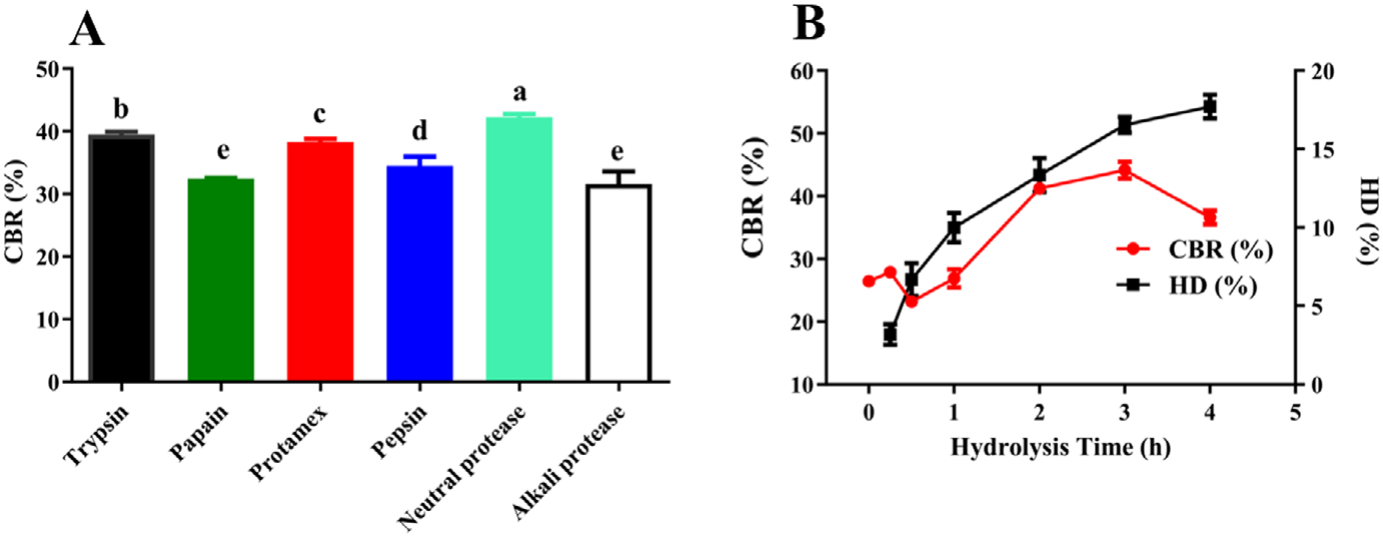
(A) CBR of LP released by different enzymes. (B) Effects of hydrolysis time on the CBR and HD of LPN. Different letters stand for significant differences (*p* < 0.05).

Figure 1A demonstrates significant differences in the CBR of LP released by various proteases. Among these, LPN exhibited the highest CBR (42.21% ± 0.54%) (*p* < 0.05). Proteases exhibit high specificity in protein hydrolysis; however, there is no clear pattern in screening proteases for preparing CBPs with potent CBR, which is generally related to the protein's origin (Gu et al. 2024). For example, double enzymes (papain and flavor enzyme) were used to prepare CBPs from sesame meal and egg white (Huang et al. 2021) and *Auxis thazard* meat (Chen et al. 2019), respectively. Hydrolysis time and HD also determine the CBR of peptides. Figure 1B indicates that the CBR of LPN increased with the increment of hydrolysis time and HD during the first 3 h but then decreased thereafter. Moderate hydrolysis enhances the CBR of peptides, but excessive hydrolysis may destroy the calcium‐binding sites of peptides, thus decreasing CBR (Wang et al. 2018; Wu et al. 2019). Therefore, a hydrolysis time of 3 h was selected for the subsequent study.

### 3.2 Single‐Factor Experiments

**FIGURE 2:**
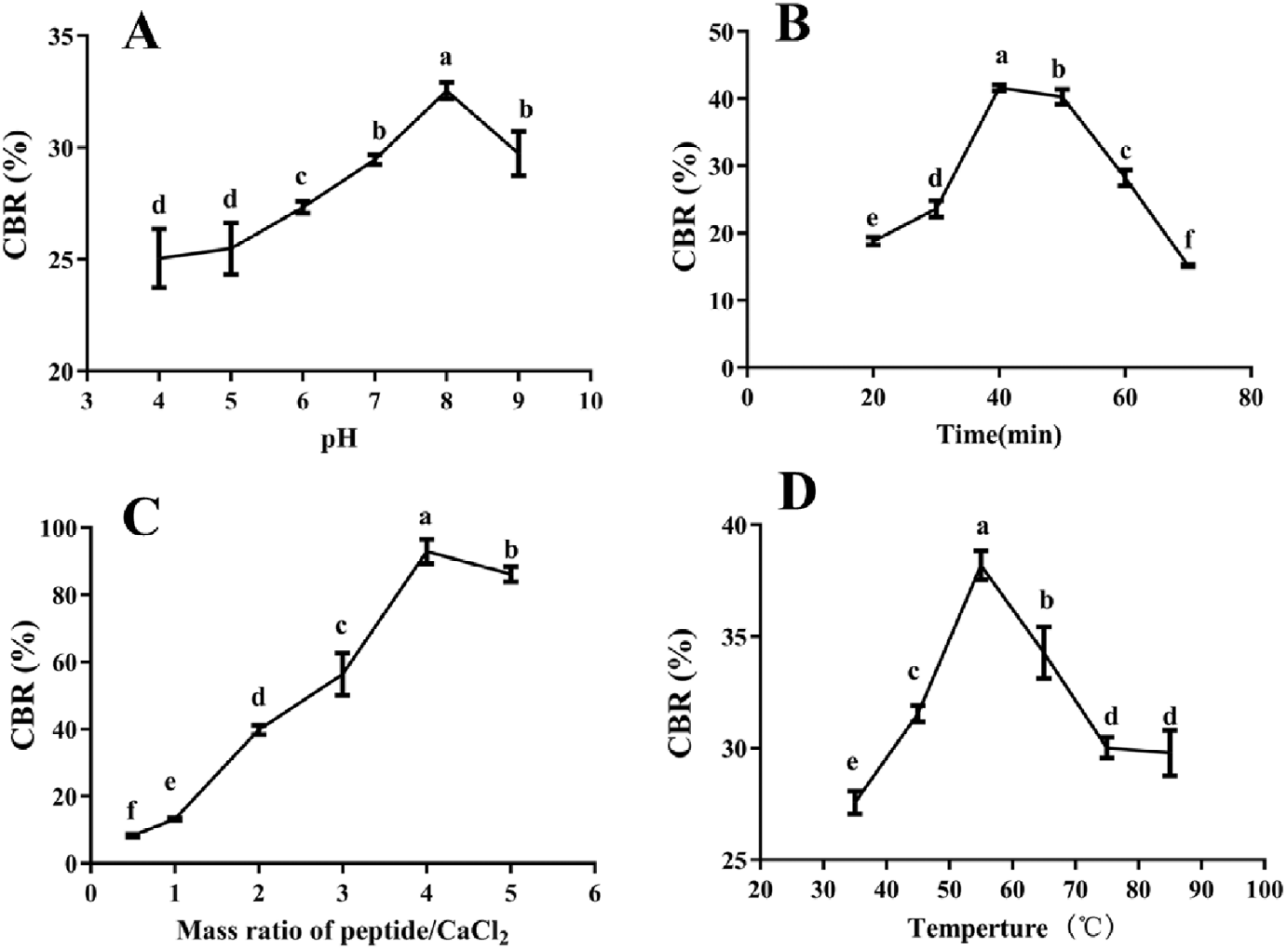
Effects of (A) pH, (B) time, (C) mass ratio of peptide/CaCl_2_, and (D) temperature on the CBR of LPN. Different letters stand for significant differences (*p* < 0.05).

**FIGURE 3:**
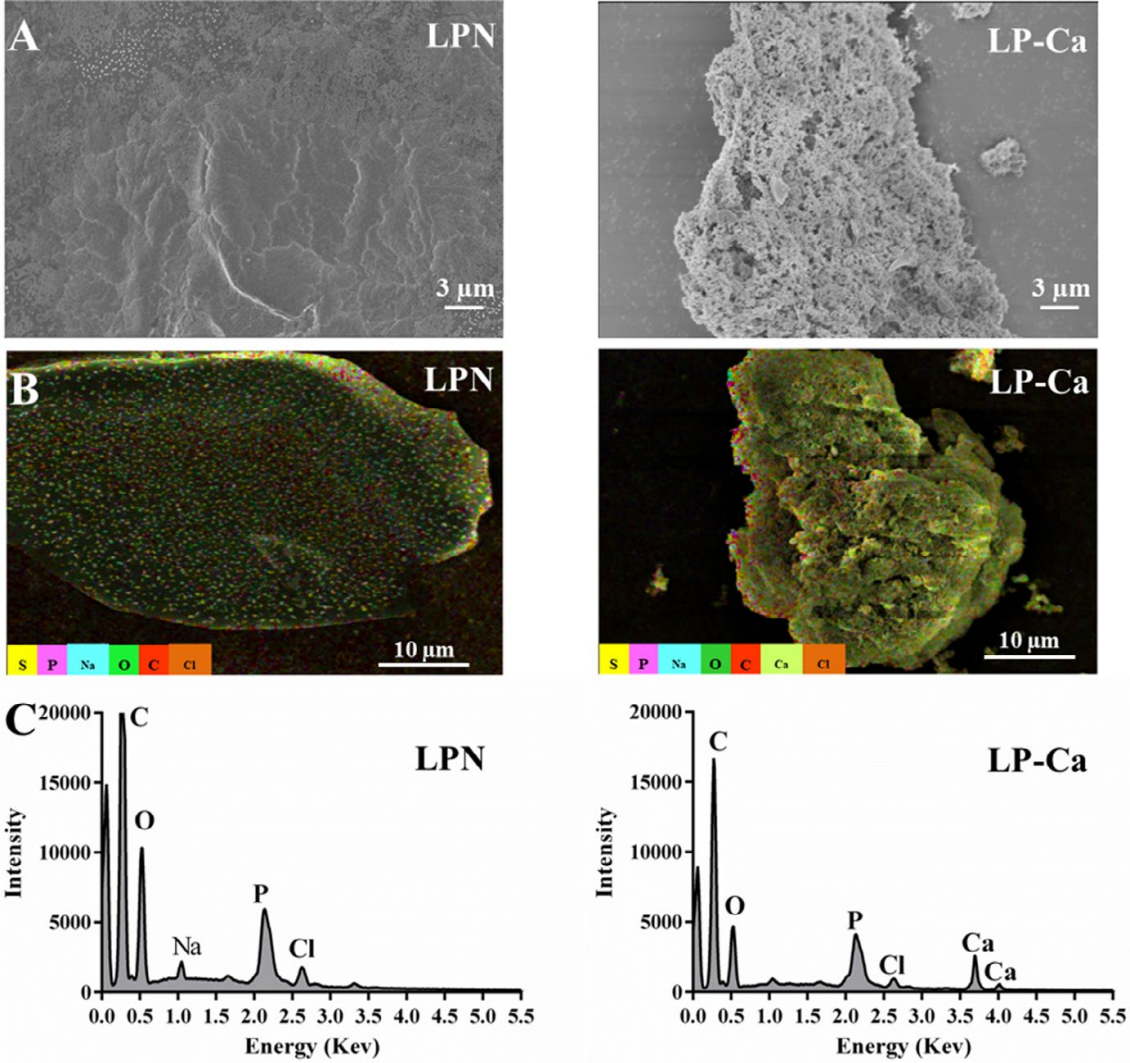
(A) Microstructure of LPN and LP‐Ca. (B) Distribution of elements and (C) element signal intensities in LPN and LP‐Ca.

Chelation conditions determine the reaction efficiency between Ca^2+^ and peptides. The CBR of LPN was highest (32.54% ± 0.37%) at a pH of 8 (*p* < 0.05) (Figure 2A). In the study of *Hericium erinaceus* peptide‐Ca, a pH8 was also selected (Gu et al. 2024). A high amount of H^+^ in low pH can occupy calcium‐binding sites in peptides, and strong alkaline conditions will consume Ca^2+^ to form Ca(OH)_2_, both of which are adverse to chelation (Wu et al. 2019). Figure 2B shows that the CBR of LPN peaked at 40 min, indicating that the chelation is a fast reaction and extending the reaction time is not beneficial (Cui et al. 2017). Sea cucumber ovum peptides also chelated with the highest amount of Ca when the reaction time was 40 min (Cui et al. 2017). Figure 3C shows that CBR was increased significantly until the mass ratio of peptides/CaCl_2_ reached 4:1, after which the CBR decreased significantly. The results indicate that adequate amounts of peptides provide sufficient binding sites for Ca^2+^ and promote chelation, but when there is an excess of peptides, some peptides do not have the opportunity to bind to Ca^2+^, thus reducing the CBR. In other peptide‐Ca chelate studies, different mass ratios of peptides/CaCl_2_ (3:1–4.5:1) are selected, indicating that it varies with the origin of peptide (Cui et al. 2017; Wu et al. 2019). Figure 2D shows that CBR topped at a temperature of 55°C (38.17% ± 0.66%). Similar results were reported in preparing pig bone collagen peptide‐Ca chelates (Wu et al. 2019). An appropriate temperature can enhance the reaction efficiency, but excessively high temperature is able to induce changes in the peptide structure, thus reducing its chelating effect with Ca^2+^ (Gu et al. 2024).

### 3.3 Optimization of LP‐Ca Preparation

BBD experiments were conducted based on single‐factor experiments. Table 1 shows the results of BBD experiments. The regression model established was *Y* = 62.54 + 4.60A + 4.64B + 10.75C + 2.67D + 4.56 AC + 4.90CD‐2.08B^2^‐4.70C^2^ + 2.60D^2^. The *p*‐value of the model was less than 0.0001 and that of lack of fit was greater than 0.05 (Table 2), implying that the model was well‐fitted and could be used to optimize LP‐Ca preparation conditions (Huang et al. 2021). The *p*‐values for A (pH), B (time), and C (Mass ratio of peptide/CaCl_2_) were less than 0.05 (Table 2), indicating that these three factors significantly affected the CBR of LPN in the reaction. The *p*‐values for AC and CD interactions were greater than 0.05, suggesting no significant interactive effects on chelation efficiency. The optimal reaction conditions, obtained using Design‐Expert software, were as follows: pH9, time 50 min, a mass ratio of peptide/CaCl_2_ 5:1, and a temperature of 65°C. Under the optimal conditions, the CBR of LPN was 88.94% ± 0.97%, surpassing the CBPs from egg white (71.93%) (Huang et al. 2021) and *Hericium erinaceus* (71.93%) (Gu et al. 2024). The calcium content in LP‐Ca was 4.23% ± 0.01%, 100 times higher than that of LPN (0.04% ± 0.01%) (Table 3), confirming the successful chelation between LPN and Ca^2+^. This value surpassed that of sheep bone collagen peptide‐Ca chelates (2.21%) (Zhang et al. 2021).

**TABLE 2:** Regression model and variance analysis of the CBR.

Source	Sum of squares	df	Mean square	*F*	*p*
Model	2414.41	9	268.27	11.80	< 0.0001
A	254.20	1	254.20	11.18	0.0034
B	258.79	1	258.79	11.38	0.0032
C	1388.04	1	1388.04	61.04	< 0.0001
D	85.48	1	85.48	3.76	0.0675
AC	83.35	1	83.35	3.67	0.0707
CD	95.93	1	95.93	4.22	0.0540
B^2^	29.18	1	29.18	1.28	0.2714
C^2^	148.80	1	148.80	6.54	0.0192
D^2^	45.36	1	45.36	1.99	0.1740
Residual	432.08	19	22.74		
Lack of fit	411.57	15	27.44	5.35	0.0584
Pure error	20.50	4	5.13		
Cor total	2846.49	28			

**TABLE 3:** Calcium content in LP‐Ca and LPN.

Sample	Calcium content (%)
LPN	0.04% ± 0.01%^a^
LP‐Ca	4.23% ± 0.01%^b^

*Note:* Different letters represent significant differences (*p* < 0.05).

### 3.4 Amino Acid Contents of LP‐Ca

Amino acid residues in peptides can provide the binding site for Ca^2+^, so their contents were analyzed (Table 4). After chelation, acidic amino acids Glu, Asp, Lys, and Ser were dramatically increased to 11.91% ± 0.01%, 11.97% ± 0.02%, 8.72% ± 0.01%, and 4.18% ± 0.03%, respectively (*p* < 0.05) (Table 4). Similarly, Glu, Asp, and Lys were enriched after *Hericium erinaceus* peptides chelating with Ca^2+^ (Gu et al. 2024). Additionally, His, Cys, and Phe were also enriched after chelation (*p* < 0.05) (Table 4). Plenty of studies have confirmed that Glu and Asp can bind to Ca^2+^ via their side‐chain carboxyl groups, and that CBPs are often rich in these two amino acids (Wu, Hu, et al. 2024). Lys can bind to Ca^2+^ with its ε‐amino nitrogen (Huang et al. 2021), and Ser is also involved in the chelation between desalted duck egg white peptides and Ca^2+^ (Hou et al. 2015). Additionally, imidazole groups from His can bind to Ca^2+^ via coordination bonds (An et al. 2022), and Cys is enriched in the egg white peptide‐Ca complex (Huang et al. 2021). Thus, previous findings supported the results herein, indicating that these enriched amino acids might be involved in the chelation with Ca^2+^.

**TABLE 4:** Amino acid contents of LPN and LP‐Ca.

Amino acids	LPN (%, W/W)	LP‐Ca (%, W/W)
Glu	10.98 ± 0.04^a^	11.91 ± 0.01^b^
Asp	8.99 ± 0.06^a^	11.97 ± 0.02^b^
Lys	8.10 ± 0.02^a^	8.72 ± 0.01^b^
Ser	4.00 ± 0.01^a^	4.18 ± 0.03^b^
His	1.07 ± 0.10^a^	1.33 ± 0.01^b^
Cys	0.21 ± 0.01^a^	0.41 ± 0.05^b^
Phe	4.58 ± 0.02^a^	4.68 ± 0.13^a^
Ala	7.32 ± 0.01^a^	6.68 ± 0.04^b^
Gly	3.29 ± 0.02^a^	3.29 ± 0.06^a^
Thr	4.35 ± 0.02^a^	3.96 ± 0.03^b^
Arg	5.33 ± 0.06^a^	4.01 ± 0.02^b^
Pro	4.54 ± 0.02^a^	3.77 ± 0.13^b^
Met	0.01 ± 0.002	Not Detected
Val	7.08 ± 0.07^a^	6.74 ± 0.03^b^
Tyr	2.96 ± 0.16^a^	2.63 ± 0.20^a^
Ile	4.19 ± 0.04^a^	3.86 ± 0.06^b^
Leu	7.01 ± 0.13^a^	5.86 ± 0.05^b^

*Note:* Different letters on the same line represent significant differences (*p* < 0.05).

### 3.5 Structural Analysis of LP‐Ca

#### 3.5.1 Microstructure and EDS Assay

The microstructures of LPN and LP‐Ca were observed. The surface of LPN was fine and smooth, displaying an intact and uniform structure (Figure 3A). Upon chelation, the microstructure became rough, loose, and porous, indicating the formation of new substances. These changes might be attributed to internal structural alterations caused by chelation between peptides and Ca^2+^ (Zhang et al. 2021). Similarly, herring egg peptide‐Ca exhibited a loose and floccule structure (Sun et al. 2020); cattle bone collagen peptide‐Ca become rougher after chelation (Zhang et al. 2021). In contrast, chicken foot peptide‐Ca displayed a firm and crystal structure after chelation, which was attributed to the aggregation induced by Ca^2+^ (Malison, Arpanutud, and Keeratipibul 2021). Thus, the morphological changes may depend on the origin of peptides. Furthermore, both EDS analysis and layered EDS image showed significant signals of Ca in LP‐Ca, while no Ca signal was found in LPN (Figure 3B,C). These results implied that Ca^2+^ successfully chelated with LPN, forming LPN‐Ca.

#### 3.5.2 UV and Fluorescence Scanning Analysis

**FIGURE 4:**
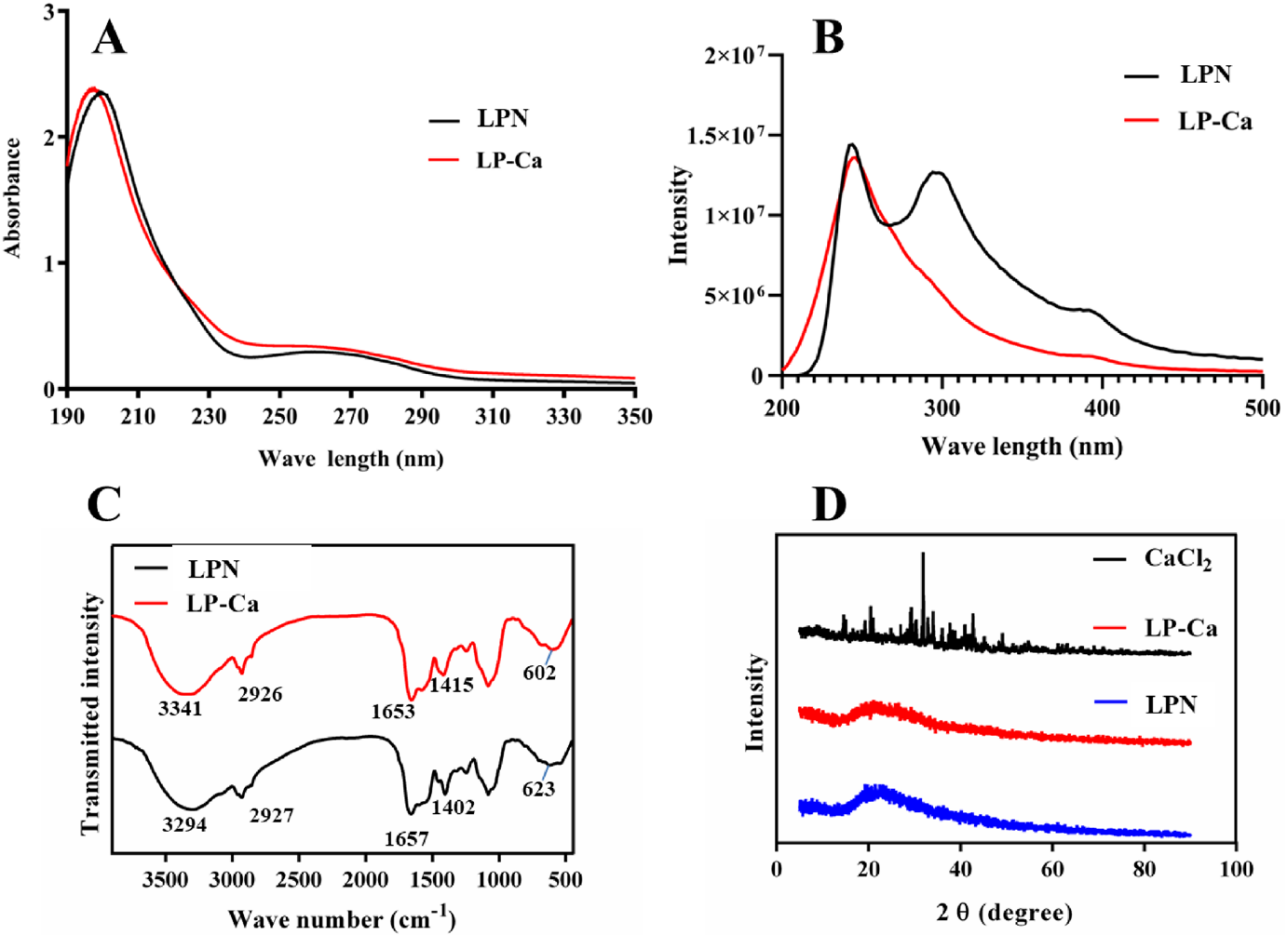
(A) UV and (B) fluorescence scanning spectra of LPN and LP‐Ca. (C) FT‐IR and (D) XRD spectra of LPN and LP‐Ca.

Possible changes of LPN after chelation were analyzed by UV and fluorescence scanning. LPN had a strong peak at 200 nm (Figure 4A), which was due to carboxyl and carbonyl in the amide bond (Huang et al. 2021). LPN also had a weak absorption peak at 258 nm (Figure 4A), caused by aromatic amino acid residues (Phe and Tyr) (Huang et al. 2021). After chelation, the strong peak moved to 195 nm with the increase of intensity (Figure 4A), suggesting the binding between Ca^2+^ and LPN. This indicates that the chelation between Ca^2+^ and LPN caused polarizing alterations of the carboxyl and the amide bond (Yang et al. 2021). In addition, the weak peak became smoother (Figure 4A), which may result from the decrease in the total contents of aromatic amino acid residues (7.54% vs. 7.31%) after chelation (Table 3).

Figure 3B shows that LPN had two fluorescence absorption peaks at 240 nm and 300 nm, respectively. The endogenous fluorescence originated from aromatic amino acids (Phe and Tyr). Upon chelation, the fluorescence intensity obviously reduced, and the absorption peak at 300 nm disappeared. The results implied that LPN folding was induced by Ca^2+^, thereby causing fluorescence quenching (Zhao et al. 2014). In addition, some chromophores (C=O) in LPN might be involved in the chelation, leading to alterations of the excited‐state energy and reduction in fluorescence strength (Zhai et al. 2023). Overall, these results further confirmed the successful chelation between LPN and Ca^2+^, and C=O and carboxyl may be involved in the chelation.

#### 3.5.3 FT‐IR and XRD Assay

FT‐IR was use to reveal the potential groups participating in the chelation of LPN‐Ca. The peak (3294 cm^−1^) of LPN was attributed to the N—H stretching vibration (Figure 4C); upon chelation, it red‐shifted to 3341 cm^−1^ (Figure 4C), which may be due to the coordination of N—H with Ca^2+^ (Wang et al. 2018). The peak at 1657 cm^−1^ of LPN resulted from the amide І band stretching vibration generated by C=O (Figure 4C) (Chen et al. 2019); upon chelation, it blue‐shifted to 1653 cm^−1^ (Figure 4C), indicating that C=O may provide binding sites for Ca^2+^. The band of LPN at 1402.41 cm^−1^ was caused by the stretching vibration of —COO— (Figure 4C) (Zhang et al. 2021); after chelation, it red‐shifted to 1415 cm^−1^ (Figure 4C), suggesting that —COO— is involved in the binding with Ca^2+^. In addition, the peak of LPN at 623 cm^−1^, caused by C—H and N—H (Huang et al. 2021), blue‐shifted to 602 cm^−1^ (Figure 4C), further implying the formation of a coordination between Ca^2+^ and N—H. Thus, —COO—, C=O, and N—H were contributed to the chelation. Huang et al. (2021) reported that carboxyl oxygen and amino nitrogen atoms were involved in the chelation between egg white peptide and Ca^2+^. Gu et al. (2024) also confirmed that —COO— and C=O groups from *Hericium erinaceus* peptides participated in the chelation. These studies support our findings.

Further, XRD analysis showed that CaCl_2_ exhibited lots of sharp and strong peaks, but these peaks were missing after reacting with LPN, leaving only a weak peak (Figure 4D). This result indicated that calcium was not simply mixed with LPN in LPN‐Ca but formed bonds with LPN, thereby changing the crystal structure of Ca^2+^ to an amorphous form. Similar results have been reported in the Crimson Snapper scale peptide‐Ca chelates (Wu et al. 2023).

### 3.6 TG and DSC Assay

**FIGURE 5:**
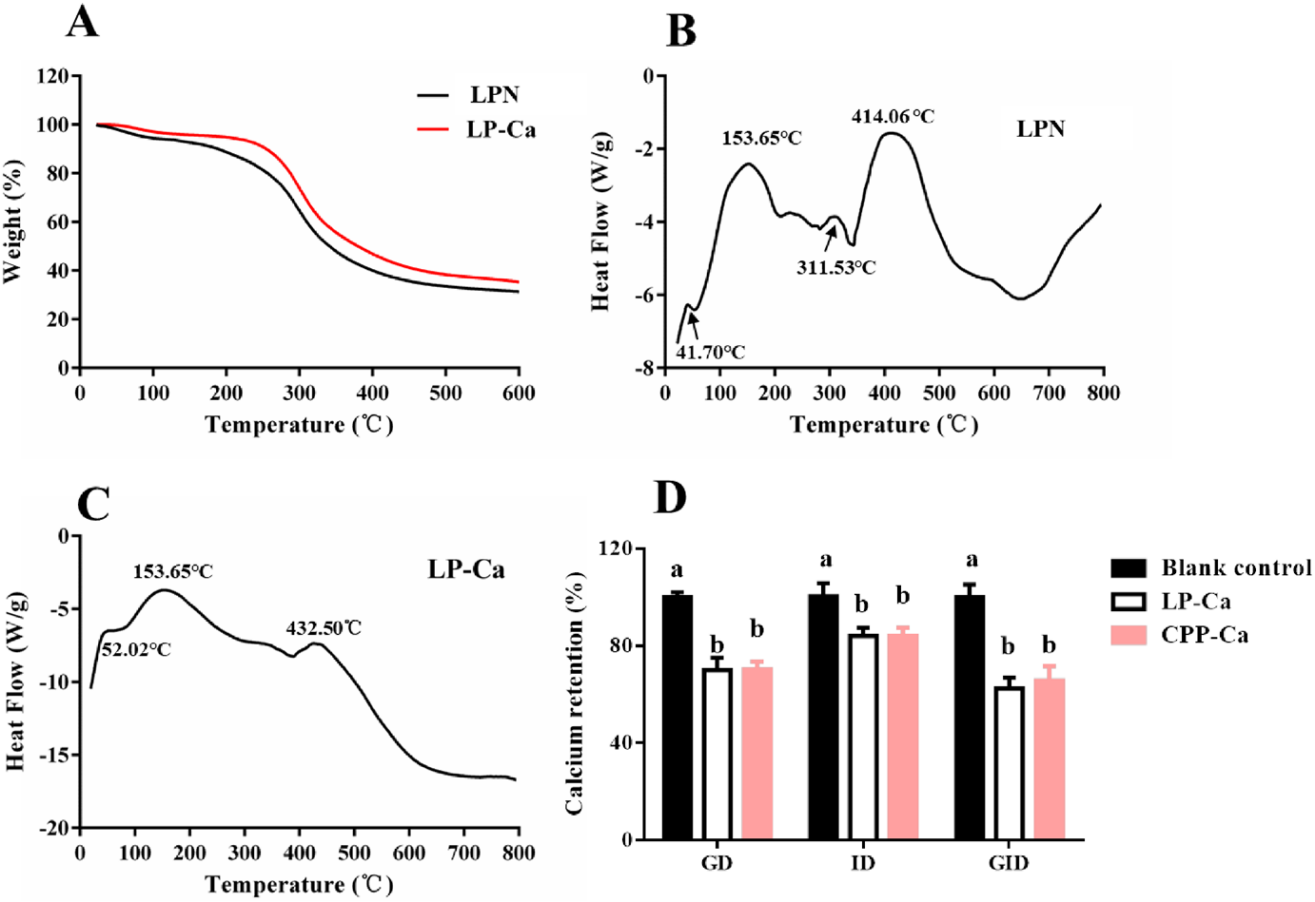
(A) TG of LPN and LP‐Ca. DSC of (B) LPN and (C) LP‐Ca. (D) Digestion stability of LPN and LP‐Ca. Different letters stand for significant differences (*p* < 0.05).

Thermal properties of LPN‐Ca were evaluated using TG and DSC assay. The weight of LPN reduced slowly from 40°C to 153°C (Figure 5A), likely due to the loss of free water and some of bound water (Gu et al. 2023). After that, the weight of LPN decreased rapidly (Figure 5A), implying the decomposition of LPN. Upon chelation, the weight loss happened at higher temperatures, suggesting a greater stability of LPN‐Ca. Moreover, LPN exhibited four strong endothermic peaks at 41.7°C, 153.65°C, 311.53°C, and 414.06°C, respectively (Figure 5B). The endothermic peaks shifted to higher temperatures upon chelation with Ca^2+^ (Figure 5C), further confirming the higher thermal stability of LP‐Ca compared to that of LPN. This may result from forming new bonds between LPN and Ca^2+^ in LP‐Ca. Similar results were reported in sheep bone collagen peptide‐Ca chelates (Wang et al. 2020) and cattle bone collagen peptide‐Ca chelates (Zhang et al. 2021).

### 3.7 Digestion Stability Assay

Once ingested, the digestive enzymes and condition will disrupt the structure of LP‐Ca, thereby leading to calcium loss and reduced bioavailability. Therefore, the digestion stability of LP‐Ca was evaluated. Approximately 30% of calcium loss occurred during the GD stage (Figure 5D) and about 10% was lost during the ID stage. The chelate was unstable in low pH environments (Figure 2A), partly due to the competitive occupation of calcium‐binding sites by excessive amount of H^+^ (Wu et al. 2019). Moreover, the hydrolyzing effects of pepsin would disrupt the original structure of LP‐Ca, causing further calcium loss (Wu, Hu, et al. 2024). The peptide‐Ca chelates from *Hericium erinaceus* are also unstable after GD, while most of Ca (more than 82%) is retained after simulated digestion (Gu et al. 2024). Nevertheless, LP‐Ca exhibited similar digestion stability with CPP‐Ca (62.33% ± 4.51% vs. 65.67% ± 6.03%), a commercial calcium supplement known for its good calcium bioavailability (Figure 5D). These findings indicate that LP‐Ca may also have good calcium bioavailability.

### 3.8 Calcium Transport Promoting Activity Assay

**FIGURE 6:**
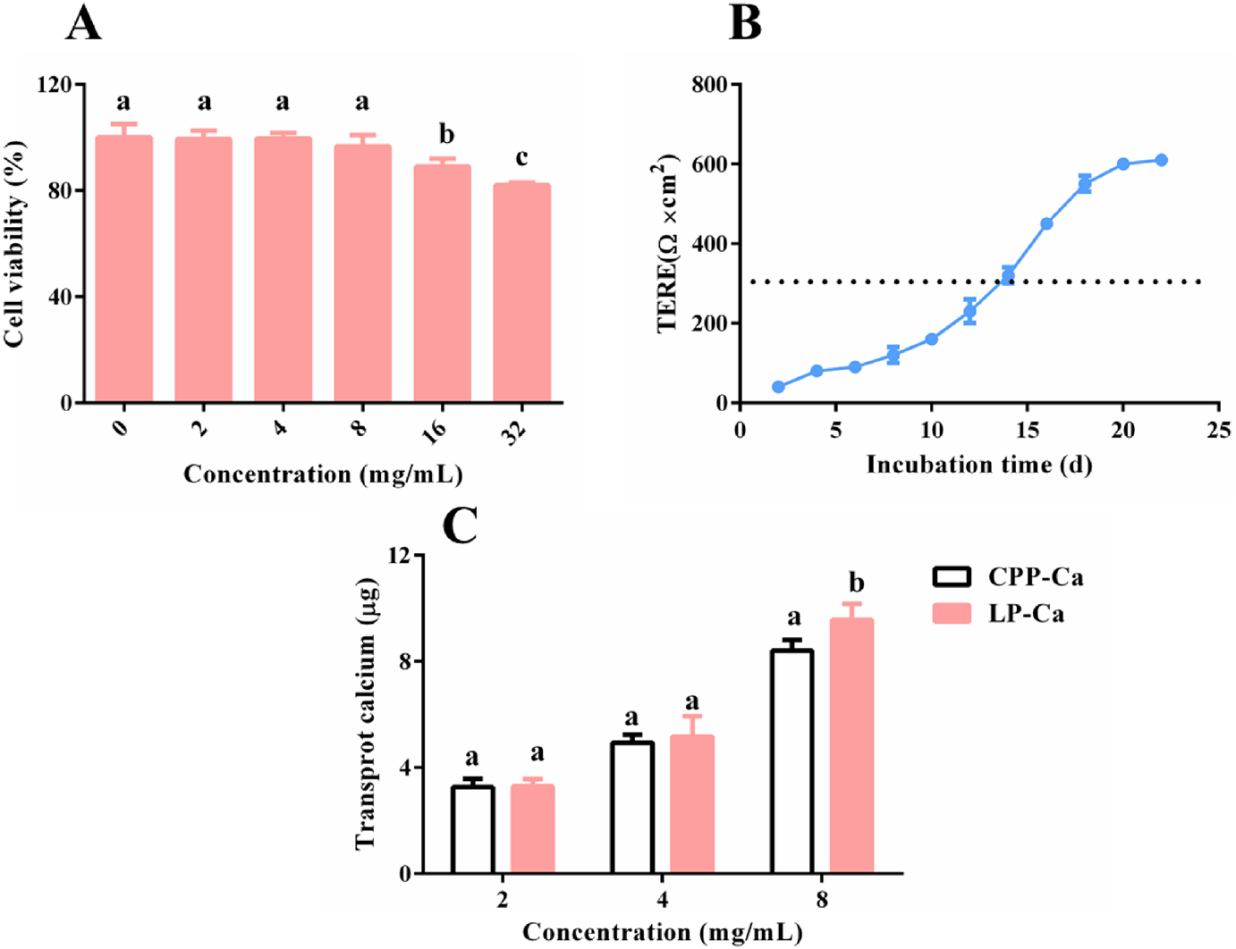
(A) Cell viability of Caco2 cells treated with LPN (0–32 mg/mL). (B) TEER value of the Caco2 monolayer at different incubation times. (C) Transported calcium of LP‐Ca and CPP‐Ca by the Caco2 monolayer model. Different letters stand for significant differences (*p* < 0.05).

To select the optimal amount of LP‐Ca for the calcium transport promoting activity assay, the viability of Caco2 cell‐treated LP‐Ca (0–32 mg/mL) was measured. Figure 6A shows that cell viability was not significantly influenced by 2–8 mg/mL of LP‐Ca; thus, this concentration range was chosen. Transcellular transport is a crucial way for the absorption of calcium ions, so a cell monolayer was established to evaluate the calcium transport promoting activity of LP‐Ca. TEER of the cell monolayer increased with extended culture time, reaching up to 300 Ω after 2 weeks (Figure 6B), implying good density and integrity of the model, thus meeting the requirements of the transporter experiment (Wu et al. 2022). Figure 6C shows that the amount of transported calcium increased from 3.30 ± 0.26 μg to 9.57 ± 0.60 μg with the increasing concentration of LP‐Ca. Moreover, the LP‐Ca group (8 mg/mL) had a higher amount of transported calcium than that of the positive group (CPP‐Ca) (*p* < 0.05). These findings indicate that LP‐Ca facilitate calcium transport, thereby enhancing the calcium bioavailability. CBPs can interact with the membrane to open the calcium channels and act as carriers for the transportation of the chelated calcium (Lin et al. 2024), thereby improving calcium absorption (Liao et al. 2020). The transcellular pathways are regulated by many proteins such as TRPV6 and Cav1.3 (Liu et al. 2018). CPP regulates the transcellular pathway by improving TRPV6 expression, thereby enhancing the transportation and absorption of Ca^2+^ (Liu et al. 2018). Additionally, peptides are also able to improve Ca^2+^ absorption by activating calcium sensing receptor, which is responsible for maintaining calcium homeostasis, transportation, and absorption (Qi et al. 2023; Wongdee et al. 2019). Herein, LP‐Ca demonstrated good calcium transport promoting activity, but the underlying mechanism for this effect remains to be elucidated.

## 4 Conclusion

This study is the first to report the preparation of LP‐Ca. LP‐Ca were prepared under the optimal conditions: pH9, time 50 min, a mass ratio of peptide/CaCl_2_ of 5:1, and a temperature of 65°C. The chelates had a rough, loose, and porous structure, and Glu, Asp, His, Cys, and Phe were enriched after chelation. —COO—, C=O, and N—H groups were contributed to the chelation. LP‐Ca demonstrated good thermal stability and retained most of calcium after simulated gastrointestinal digestion. LP‐Ca are promising calcium supplements with promoting effects on calcium transport.

## Author Contributions

**Haofeng Gu:** conceptualization (lead), data curation (equal), funding acquisition (equal), supervision (equal), writing – original draft (equal), writing – review and editing (equal). **Lei Liang:** investigation (equal), supervision (equal). **Yang Wei:** investigation (equal), methodology (equal). **Jia Hao Wang:** investigation (equal), methodology (equal). **Wanning Ma:** investigation (equal), validation (equal). **Yuyu Fu:** investigation (equal), validation (equal). **Dan Fan:** investigation (equal), validation (equal). **Wanxiang Gao:** investigation (equal). **Jiayao Yang:** investigation (equal). **Xinyu Zheng:** investigation (equal). **Tingshu Chen:** investigation (equal). **Yuexin Chen:** investigation (equal).

## Conflicts of Interest

The authors declare no conflicts of interest.

## Data Availability

The data that support the findings of this study are available from the corresponding author upon reasonable request.
